# Reconfigurable Yagi-Uda antenna based on a silicon reflector with a solid-state plasma

**DOI:** 10.1038/s41598-017-17425-8

**Published:** 2017-12-08

**Authors:** Da-Jin Kim, Jang-Soon Park, Cheol Ho Kim, Jae Hur, Choong-Ki Kim, Young-Kyun Cho, Jun-Bong Ko, Bonghyuk Park, Dongho Kim, Yang-Kyu Choi

**Affiliations:** 1 School of Electrical Engineering, Korea Advanced Institute of Science and Technology, (KAIST) 291 Daehak-ro, Yuseong-gu, Daejeon 34141 Republic of Korea; 2 Deparment of Electrical Engineering, Sejong University, 209 Neungdong-ro, Seoul, 05006 Republic of Korea; 3 Mobile RF Research Section, Electronics and Telecommunications Research Institute, 218 Gajeong-ro, Yuseong-gu, Daejeon 34129 Republic of Korea

## Abstract

This paper describes the fabrication and characterization of a reconfigurable Yagi-Uda antenna based on a silicon reflector with a solid-state plasma. The silicon reflector, composed of serially connected p-i-n diodes, forms a highly dense solid-state plasma by injecting electrons and holes into the intrinsic region. When this plasma silicon reflector is turned on, the front-realized gain of the antenna increases by more than 2 dBi beyond 5.3 GHz. To achieve the large gain increment, the structure of the antenna is carefully designed with the aid of semiconductor device simulation and antenna simulation. By using an aluminum nitride (AlN) substrate with high thermal conductivity, self-heating effects from the high forward current in the p-i-n diode are efficiently suppressed. By comparing the antenna simulation data and the measurement data, we estimated the conductivity of the plasma silicon reflector in the on-state to be between 10^4^ and 10^5^ S/m. With these figures, silicon material with its technology is an attractive tunable material for a reconfigurable antenna, which has attracted substantial interest from many areas, such as internet of things (IoT) applications, wireless network security, cognitive radio, and mobile and satellite communications as well as from multiple-input-multiple-output (MIMO) systems.

## Introduction

Recently, a reconfigurable antenna (RA) has received substantial interest from many areas, such as internet of things (IoT) applications, wireless network security, cognitive radio, and mobile and satellite communications as well as from multiple-input-multiple-output (MIMO) systems^1–7^. These areas need various types of antennas with different radiation patterns, polarization, bandwidth, or operating frequencies to efficiently cope with numerous commercial services^8^. However, inserting multiple antennas into a single device is challenging because of the limited space and high cost. In that respect, a reconfigurable antenna that can replace multiple antennas is very attractive in those areas. There are many different forms of reconfigurable antenna, such as an antenna using a varactor, a micro-electro-mechanical systems (MEMS) switch, a reconfigurable feeding network, an array structure, or tunable material^9–13^. Among them, tunable material has a big potential applicability because antenna properties can be changed in many different ways: for example, selection of the material as a reflector, control of the length in the radiation part, and modification of the geometry of the antenna.

An example of the reconfigurable antenna using a tunable material is a plasma antenna. In a gas-phase plasma antenna, an ionized gas plasma formed in a discharge tube by bursts of applied RF power is used as an alternative to metal conductors^14,15^. By turning on and off the plasma, the antenna is dynamically reconfigured without the aid of RF elements. The plasma antenna also has a stealth property in the off-state because the antenna is reverted to a dielectric-phase, which is invisible to the radar^16^. In a solid-state plasma antenna, known as a plasma silicon antenna, a cloud of electrons and holes replaces the metal conductors^17^. The density of the electron-hole plasma can be controlled by light illumination, thermal heating, and voltage biasing with a wide range^18–22^. Hence, conductivity (*σ*_*si*_) of the silicon can be intentionally modulated. It is inferred that the silicon provides superior tunability to the plasma antenna by providing the electron-hole plasma with a high density^23,24^. Compared to the gas-phase plasma antenna, the plasma silicon antenna has advantages in price and size. By employment of matured and commercial silicon-based fabrication technology, a confined area of the solid-state plasma with a tunable *σ*_*si*_ can be precisely controlled with the aid of photolithography and ion implant. Moreover, the silicon technology has the potential to provide low cost and mass production with high packing density by integrating DC-bias lines, RF-feed, and the control circuit with the plasma silicon antenna.

Herein, to verify silicon as a tunable material for a reconfigurable antenna, we selected the structure of a commonly used Yagi-Uda antenna for its simple structure and high gain^25^. The Yagi-Uda antenna is a directional antenna composed of a simple driving element, a reflector, and directors^26^. High gain in a specific direction, which is a measure of the amount of electromagnetic (EM) energy received in the specified direction, is required in numerous practical applications^27–31^. The reflector enhances the gain by reflecting the radiated wave toward the forward direction while preventing backward radiation. If the reflector is replaced by silicon with high tunability of *σ*_*si*_, the antenna gain is accordingly modulated. Therefore, the Yagi-Uda antenna using a silicon-based reflector is a simple and effective structure to demonstrate reconfigurability of the antenna. In our previous study, the feasibility of a primitive Yagi-Uda antenna with a silicon-based reflector was preliminarily reported^32^. The Yagi-Uda antenna was turned on and off by biasing the DC voltage. Although it showed a gain difference between the on-state and off-state of the silicon-based reflector, it suffered from a small gain difference as well as a burning issue arising from a poor *σ*_*si*_, the self-heating effect, and radiation interference among adjacent DC-bias lines.

In this paper, we demonstrate a revamped reconfigurable Yagi-Uda antenna using a plasma silicon reflector for resolving the abovementioned problems and optimizing the antenna. We provide a guideline for designing the reconfigurable Yagi-Uda antenna with the plasma silicon reflector with the aid of numerical simulations, electromagnetic (EM) analysis, and experiments. A ceramic and polymer based printed circuit board (PCB) called the RF-35 substrate, which has been widely and commercially used, was replaced with an aluminum nitride (AlN) substrate that created heat-sink mitigating self-heating effects owing to its high thermal conductivity^33^. The size of the antenna is also reduced due to the high permittivity (ε_AlN_ = 8.56 ε_0_) of the substrate. Prior to fabricating the Yagi-Uda antenna, we optimized its structural parameters by combining the simulation data from the semiconductor device simulator (SILVACO)^34^ and from the antenna simulator (Ansys HFSS software)^35^ to minimize the power consumption and interference among adjacent DC-bias lines. The reflector in the Yagi-Uda antenna was comprised of serially connected p-i-n diodes (diode with a wide intrinsic (i) semiconductor region between a p-type doped region (p) and a n-type doped region(n)), which could be turned on and off to control *σ*_*si*_ of the reflector. By analyzing the antenna reflection coefficients and radiation properties, we experimentally demonstrated that the plasma silicon reflector was successfully tunable from an insulator to metal and vice versa. To the best of our knowledge, our group showed the measured gain of the fabricated plasma silicon antenna for the first time in our previous research. Herein, we demonstrate the full-functionality of the fabricated plasma silicon antenna, which includes both electrical properties of the p-i-n diode and the measured as well as simulated antenna characteristics.

## Results and Discussion

**Figure 1:**
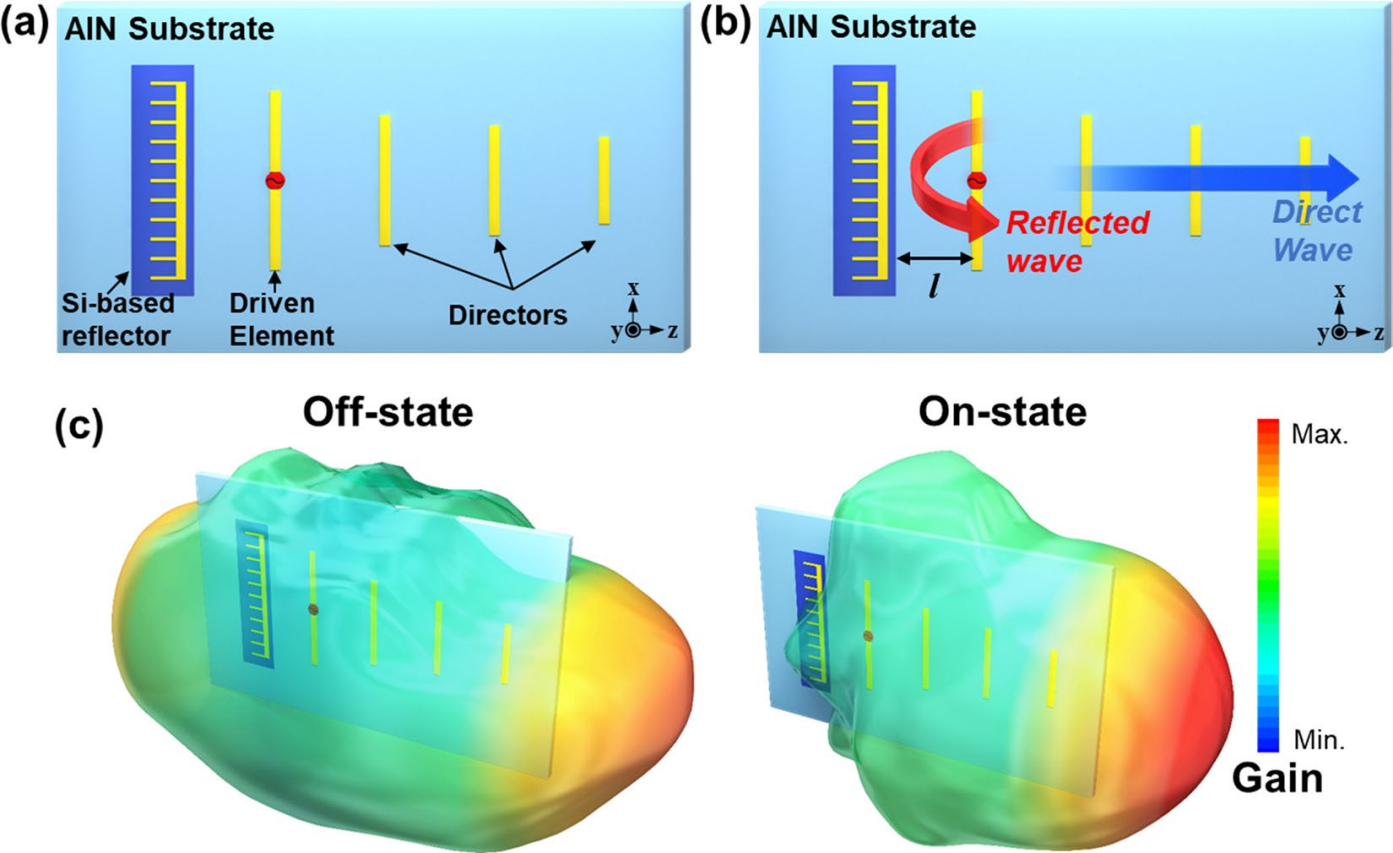
(**a**) The geometry and (**b**) operational principle of the proposed reconfigurable Yagi-Uda antenna using the plasma silicon reflector. (**c**) Conceptual 3-dimensional (3D) radiation patterns of the proposed antenna when the plasma silicon reflector is in the off-state or on-state.

A conceptual operation of the proposed reconfigurable Yagi-Uda antenna is shown in Fig. 1. The overall geometry of the proposed reconfigurable Yagi-Uda antenna with a reconfigurable plasma silicon reflector, a monopole feeder, and three directors is shown in Fig. 1(a). A commercial AlN substrate with a dielectric constant of 8.56 has been chosen for its high thermal conductivity to efficiently dissipate heat generated from the plasma silicon reflector.

The operational principle of the proposed reconfigurable Yagi-Uda antenna is illustrated in Fig. 1(b). To obtain high antenna gain in the targeted positive z-direction, the wave reflected by the reflector must interfere constructively with the wave launching from the monopole driven element. In other words, they must be in the same phase at the position of the driven element, which is formulated by

1 $${\phi }_{R}-2\beta l=2N\pi $$

where *φ*_*R*_ is the reflection phase of the reflected wave, *β* is the effective phase constant, and *N* is a positive integer. Here, −2*βl* denotes the overall phase delay for the round-trip distance of 2*l*. The reflected wave is affected by the conductivity of the reflector, *σ*_*si*_. A higher *σ*_*si*_ provides a stronger reflection, which is desirable for obtaining a high gain in the positive z-direction. When the plasma silicon reflector is in the on-state, the antenna gain, which strongly depends on *σ*_*si*_, drastically increases. For easier understanding of the role of the reflector, conceptual 3-dimensional (3D) radiation patterns of the proposed antenna are shown in Fig. 1(c). To emphasize the effect of the reflector, these patterns are trimmed based on the result of the antenna simulation (refer to Supplementary Information S1). From these data, the influence of the reflector on the radiation property of the antenna is clearly confirmed.

**Figure 2:**
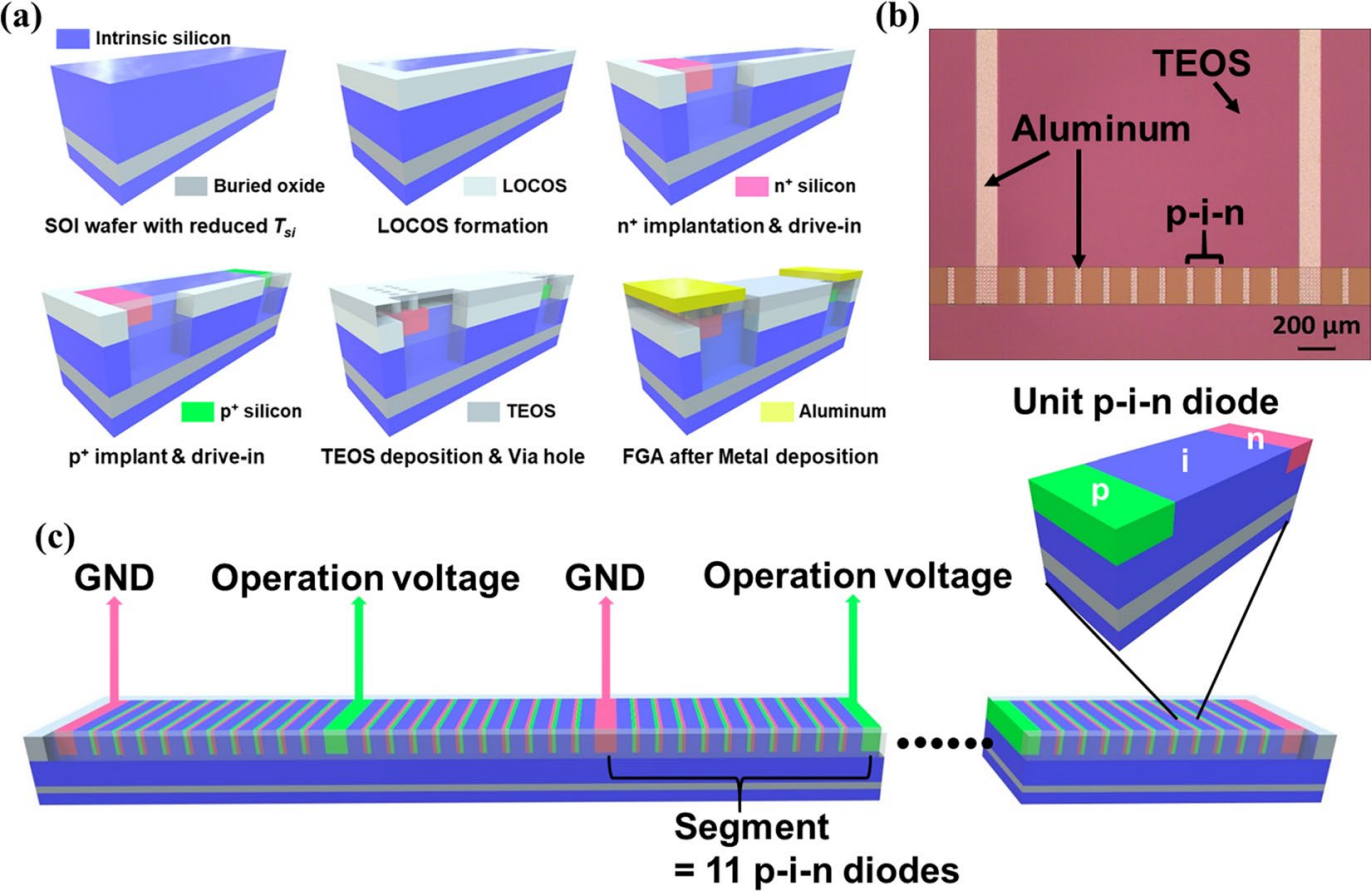
(**a**) The fabrication process of the unit p-i-n diode. (**b**) The optical photograph of the fabricated p-i-n diode array. (**c**) The structure of serially connected p-i-n diodes, and the bias configuration of the array of diodes used to construct the plasma silicon reflector.

One of the distinct features of the proposed Yagi-Uda antenna compared to a conventional Yagi-Uda antenna is that the reflector is fabricated using commercial silicon technology. To reflect the wave launched from the driven element, the reflector should have a *σ*_*si*_ as high as possible. Herein, to achieve a high *σ*_*si*_, serially connected p-i-n diodes are adopted. The *σ*_*si*_ of intrinsic region in the p-i-n diode can be controlled by injection of electrons and holes from the n-type and p-type heavily doped region to the intrinsic region by applying forward voltage between the p-type region and the n-type region. As shown in Fig. 2(c), serially connected p-i-n didoes are divided into several segments to control the operation voltage. Each segment shares the same operation voltage with the ground voltage of an adjacent segment. In other words, segments are connected in parallel, and the source of leakage current is inherently removed without use of any isolation technology, such as local oxidation of silicon (LOCOS) or shallow trench isolation. The fabrication process of the unit p-i-n diode is described in Fig. 2(a). First, to make the SOI wafer, one intrinsic wafer was bonded to another intrinsic wafer with a 1-μm oxide layer on the top, which is called buried oxide. It is desirable to have a top silicon wafer with its doping level as low as possible because a low doping level in the intrinsic region produces both a low *σ*_*si*_ in the off-state and a longer lifetime for carriers owing to a lower recombination rate^36^. Next, the top silicon of the bonded SOI wafer was ground to a thickness of 5 μm. When the thickness of the top silicon (*T*_*si*_) is thinner than 5 μm, poor uniformity of the top silicon arises from the limitation of the chemical mechanical polishing (CMP) process, which affects the device yield. Since a thickness of 5 μm is enough to reflect the incident wave, we can minimize the operating power (refer to Supplementary Information S2). In addition, the bottom silicon should be intrinsic silicon and have a *σ*_*si*_ lower than 0.01 S/m. Otherwise, the portion of incident wave is always reflected by the bottom silicon (refer to Supplementary Information S3). Then, the LOCOS process was conducted to isolate the active area from the dummy area. Afterwards, a highly n-typed doped (n^+^) junction was formed by phosphorus implantation with energy of 80 keV and a dose of 10^16^ cm^−2^. To make the junction deep, a drive-in process was carried out. Similarly, after the highly p-typed doped (p^+^) junction was formed by boron implantation with energy of 80 keV and a dose of 10^16^ cm^−2^, the drive-in process was applied once again. Two drive-in processes are designed to make the p-type junction depth and the n-type junction depth the same: 1 μm. In the next step, tetraethyl orthosilicate (TEOS) was deposited by low-pressure chemical vapor deposition (LPCVD) to passivate the device and to make a via hole before metal deposition. Finally, forming gas annealing (FGA)^37,38^ was conducted to passivate the interface between the TEOS and top silicon after the aluminum deposition via the sputtering process. An optical photograph of the completed plasma silicon reflector is shown in Fig. 2(b). After that, the bottom silicon of the fabricated plasma silicon reflector was ground to 63 μm to make the positon of the reflector comparable to the driven element before it was attached to the AlN substrate. Finally, the implanted bias lines on the substrate were connected to the metal pad of the plasma silicon reflector with gold wires.

**Figure 3:**
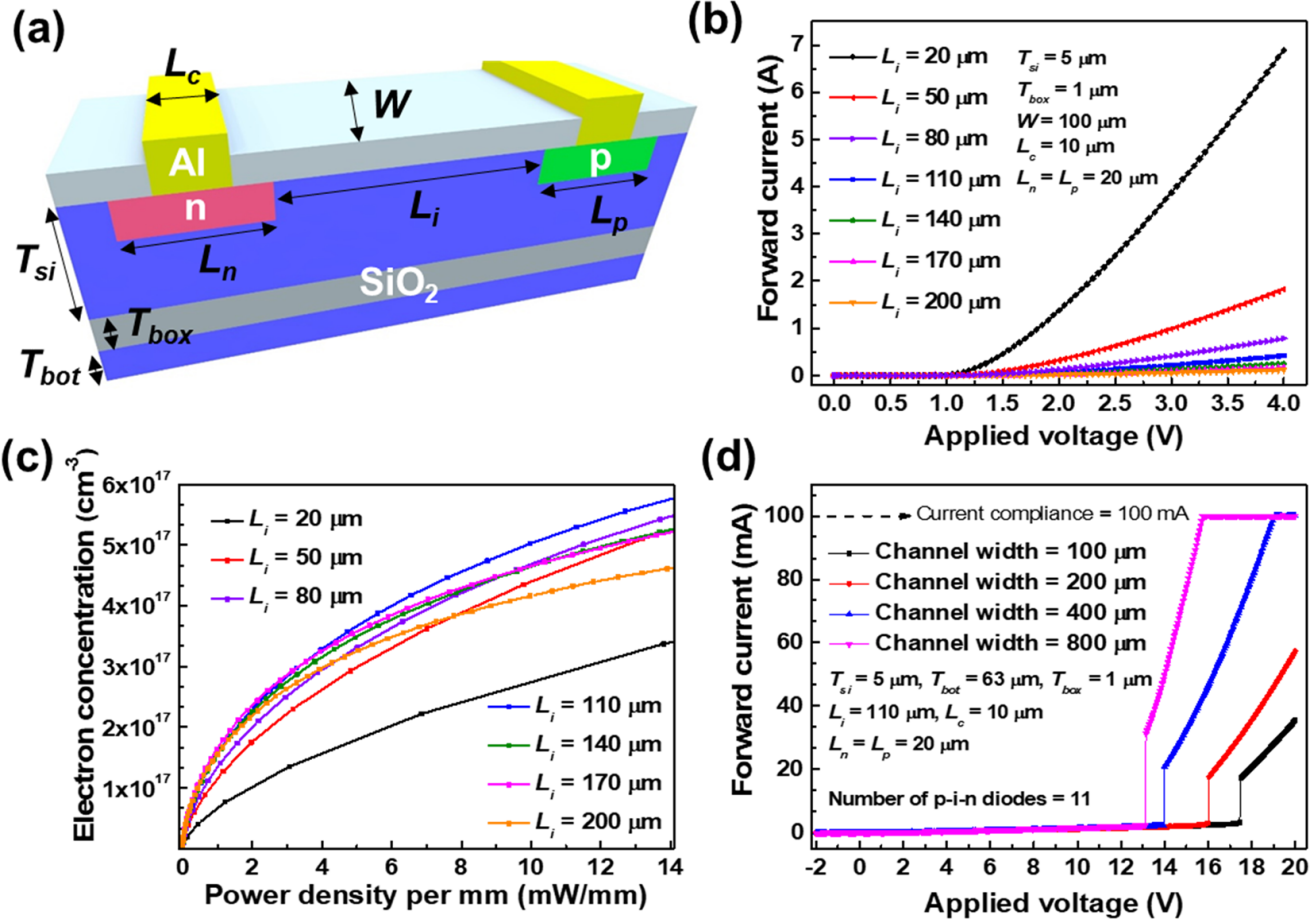
The measured and simulated electrical characteristics of the plasma silicon reflector (**a**) The geometry of the structural parameters in the unit p-i-n diode. (**b**) Simulated I-V curves of the unit p-i-n diode according to *L*_*i*_. (**c**) Simulated curves of the electron concentration versus the power density according to *L*_*i*_. (**d**) Measured I-V curves of the serially connected p-i-n diodes according to *W*.

In the plasma silicon reflector, there are many device parameters to be optimized as shown in Fig. 3(a). We carefully conducted numerical simulations to optimize those parameters with the aid of the SILVACO Atlas. The contact length (*L*_*c*_), the thickness of the buried oxide (*T*_*box*_), the length of the p-typed heavily doped region (*L*_*p*_), and the length of the n-type heavily doped region (*L*_*n*_) are insensitive process parameters, which do not significantly affect the electrical characteristic when they are large enough. Therefore, they were fixed as follows: *L*_*c*_ is 10 μm, *T*_*box*_ is 1 μm, and both *L*_*p*_ and *L*_*n*_ are 20 μm. However, the intrinsic channel length (*L*_*i*_) and *T*_*si*_ are key parameters that notably influence the reflector characteristics. Figure 3(b) shows the forward current versus the applied voltage characteristics of a unit p-i-n diode according to the *L*_*i*_. Below an applied voltage of 0.7 V, the forward currents with various *L*_*i*_ are similar, but beyond an applied voltage of 0.7 V, the p-i-n diode with a lower *L*_*i*_ exhibits a higher forward current due to a limited diffusion length and a decreased channel resistance^39^. From the measured current, the electron concertation, which is proportional to *σ*_*si*_, is extracted according to the applied voltage for each *L*_*i*_. Consideration of the electron concentration solely is enough to characterize *σ*_*si*_ of the p-i-n diode because the hole concentration is the same as the electron concentration with the opposite polarity. The difference between electrons and holes is that electrons are injected from the n-type heavily doped region and holes are injected from the p-type heavily doped region. From the electron concertation versus applied voltage curves and I-V curves of the p-i-n diode, the change in the electron concentration according to the power density (mW/mm) for each *L*_*i*_ was calculated as shown in Fig. 3(c). The p-i-n diode shows semi-metallic characteristics with a power consumption of a few milliwatts per millimeter. Since a *L*_*i*_ of 110 μm has the highest electron concentration at the given power density, *L*_*i*_ was fixed at 110 μm. Measured I-V characteristics from a single segment, which is composed of 11 p-i-n diodes, are shown in Fig. 3(d). Beyond the latch-up voltage, the forward current is abruptly increased because all the p-i-n diodes are turned on simultaneously^40^. This latch-up behavior is useful for the selection of the appropriate operation voltage. *σ*_*si*_ dramatically changes above the latch-up voltage. The appropriate operation voltage of the plasma silicon reflector during the antenna measurement in an anechoic chamber was set at 16 V with the consideration of the latch-up voltage. The channel width (*W*) of the p-i-n diode varied from 100 μm, 200 μm, and 400 μm to 800 μm. The forward current was linearly proportional to *W*. To increase the antenna gain, a wider *W* was preferred (refer to Supplementary Information S4). Finally, the optimized device parameters were *T*_*si*_ of 5 μm, *L*_*i*_ of 110μm, and *W* of 800 μm.

**Figure 4:**
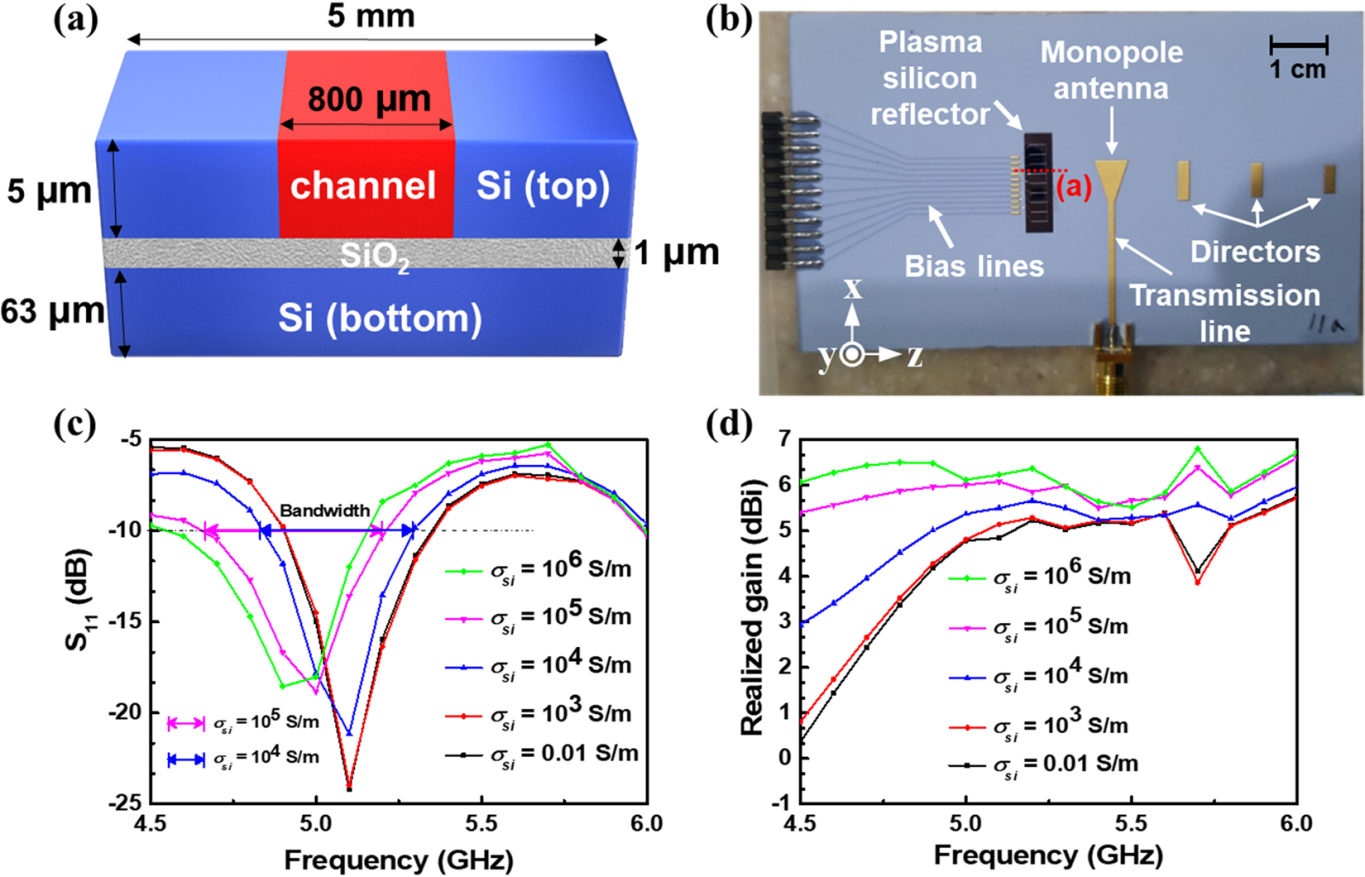
(**a**) The cross-sectional view of the simulated antenna. (**b**) The simulated S_11_ according to *σ*_*si*_. (**c**) The simulated gain in the positive z-direction.

The cross-sectional view of the plasma silicon reflector is shown in Fig. 4(a). To design the antenna precisely, the actual distribution of carrier concertation in the channel region in the on-state should carefully be considered in antenna simulation. Therefore, the carrier concentration along the vertical direction and the lateral direction of the intrinsic region in the on-state was investigated through numerical simulation (refer to Supplementary Information S5). The carrier concentration of 5 × 10^17^ cm^−3^ was uniformly distributed along the vertical direction at a *L*_*i*_ of 110 μm. The carrier concentration of 5 × 10^17^ cm^−3^ was also almost uniformly distributed along the lateral direction at the same *L*_*i*_ of 110 μm.

The overall shape of the fabricated antenna is shown in Fig. 4(b), which consists of a driving monopole antenna, 3 directors, the plasma silicon reflector, a microstrip transmission line, and 11 DC-bias lines. All metal patterns: the monopole antenna, the directors, the micro strip transmission line, and the DC-bias lines, were patterned by industrial silk screen printing^41^. Due to an adhesion problem, the metal patterns were composed of gold/nickel/silver on a 1-mm-thick commercial AlN substrate, which served as the heat sink. The micro strip transmission line was connected to a 50 Ω coaxial cable. To evaluate the antenna characteristics of the fabricated antenna, the antenna input reflection coefficient (S_11_) and the antenna realized gain (antenna gain including the amount of the EM energy loss reflected back to the source due to the impedance mismatch at the input port of the antenna) were primarily characterized. Supportive simulations were carried out for a study of the impedance matching property according to *σ*_*si*_, as shown in Fig. 4(c). From this, it is inferred that S_11_ is insensitive to *σ*_*si*_. When *σ*_*si*_ increased from 10^4^ to 10^5^ S/m, the impedance matching bandwidth in which S_11_ was lower than −10 dB, also widened approximately from 450 MHz to 550 MHz, which corresponded to a fractional bandwidth of 8.86% and 11.16%. The simulated realized gain in the positive z-direction according to *σ*_*si*_ is also displayed in Fig. 4(d). The realized gain is proportional to *σ*_*si*_, which is far more sensitive to *σ*_*si*_ than S_11_.

**Figure 5:**
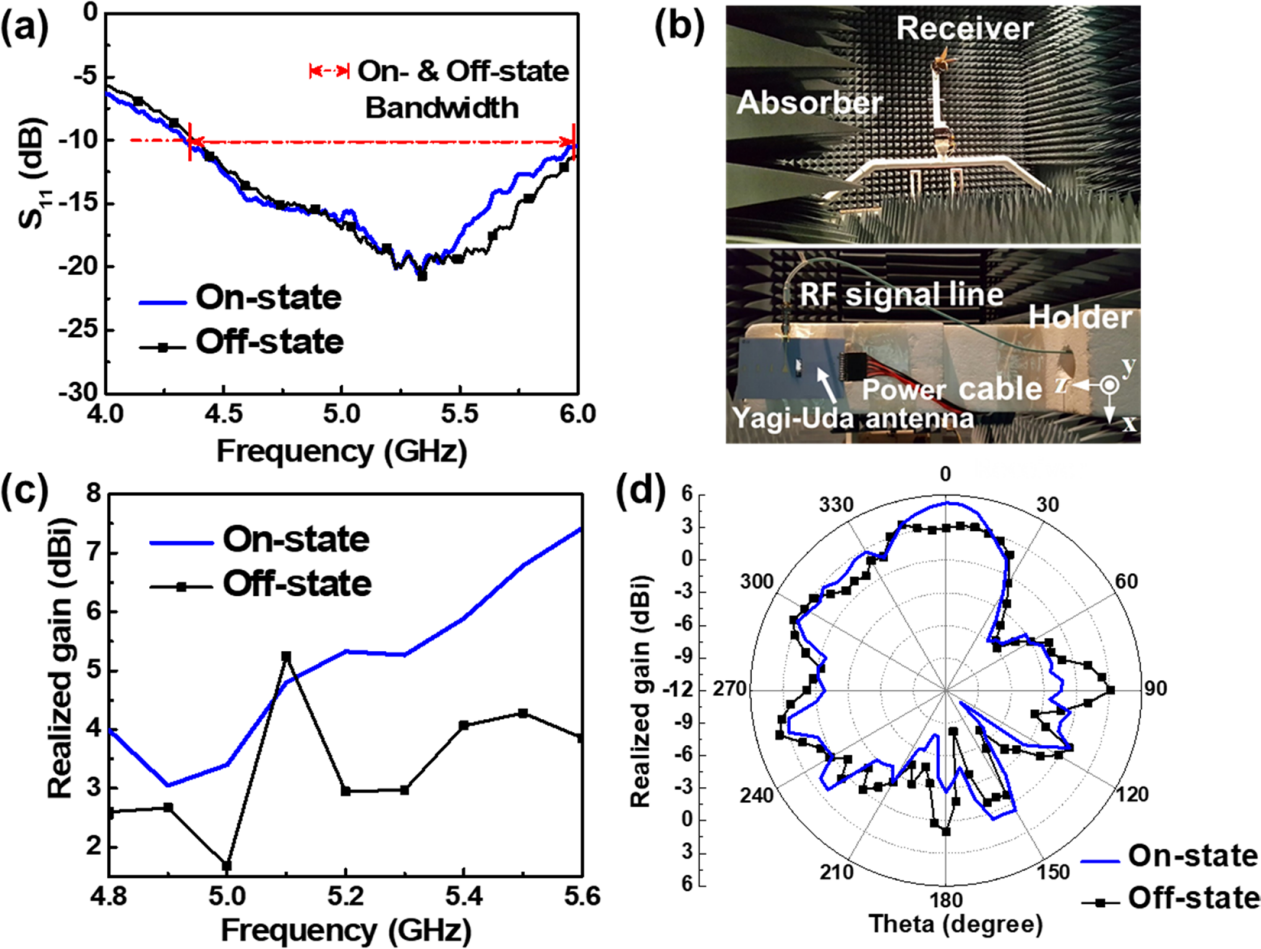
The effect of switching states on the proposed plasma silicon reflector (**a**) S_11_. (**b**) The gain in the positive z-direction. (**c**) 2-dimenstional (2D) radiation patterns at 5.3 GHz. (**d**) A photograph of a fully anechoic chamber with the receiving horn antenna and the fabricated Yagi-Uda antenna on a commercial rohacell holder.

The measured antenna characteristics: S_11_ and radiation properties, are presented in Fig. 5, which reveals that the impedance matching bandwidth is approximately 1.5 GHz covering from 4.5 to 6 GHz, which corresponds to a fractional bandwidth of 28.57%.

The measurement environment is described in Fig. 5(b). Both the receiver and the Yagi-Uda antenna are located in an anechoic chamber whose inner wall is composed of the absorber. Figure 5(c) shows how the realized gain in the positive z-direction varies when the plasma silicon reflector is turned on and off. When the plasma silicon reflector is turned on, the realized gain increases more than 2 dBi beyond 5.3 GHz. At 5.1 GHz, the realized gain is higher in the off-state, which might be due to the many gold wires connecting the plasma silicon reflector to the DC-bias lines (refer to Supplementary Information S6). Comparing resonant frequencies in Figs 4(c) and 5(a) reveals that the measured S_11_ has shifted up about 400 MHz toward the high frequency region. A similar frequency shift can also be found from the realized gain behaviors shown in Figs 4(d) and 5(c). From the realized gain shift, it is important to notice that *σ*_*si*_ can be estimated as between 10^4^ and 10^5^ S/m. The radiation pattern on an E-plane containing a radiating electric field vector, which was measured at 5.3 GHz is shown in Fig. 5(d). As the plasma silicon reflector is turned on, the front-realized gain (at 0°) increases and the rear-realized gain (at 180°) decreases, which proves the proposed silicon reflector well mimics a metallic conductor.

In our study, the silicon technology was used to make the Yagi-Uda antenna reconfigurable. The silicon material exhibited good tunability by forming a highly dense solid-state plasma in the intrinsic channel region of the p-i-n diode. When the plasma silicon reflector was in the on-state, the realized gain in the positive z-direction increased more than 2 dBi beyond 5.3 Ghz compared to the off-state. A large gain increment was achieved by structure optimization with the device and antenna simulator and self-heating relaxation with the AlN substrate. From measuring the characteristic of the fabricated Yagi-Uda antenna in an anechoic chamber, we estimated the *σ*_*si*_ in the on-state to be between 10^4^ and 10^5^ S/m. This paper is the first to demonstrate full-functionality of the plasma silicon antenna, which includes the properties of the silicon device and the measured characteristics of the fabricated antenna. Our results confirm that the silicon material with its technology has a potential to be directly applied to a reconfigurable antenna as a radiating element or entire antennas in the future.

## Methods

### Fabrication of the antenna

The SOI wafer was fabricated by a commercial wafer bonding technology. Forming p-i-n diodes on the SOI wafer was conducted through a commercial service of silicon technology. The Yagi-Uda antenna was fabricated on the AlN substrate with metal patterns made by industrial silk screen printing. (Y.TECH, Co.) Rn2 technologies, Co. provided the AlN substrate. By silk screen printing, a silver layer with a thickness of 8 μm was deposited and patterned on the AlN substrate. Solider resistor was also deposited on the silver patterns. The electroless plating was conducted to deposit a nickel layer with a thickness of 3 μm and a gold layer with a thickness of 0.1~0.2 μm. All interconnections were carried out by the commercial company. (RUATECH Inc.).

### Characterization

All electrical measurements were carried out without any device encapsulation. Electrical measurements were carried out using a HP4156 semiconductor parameter analyzer under ambient conditions. The antenna input reflection coefficient (S_11_) was measured by Vector Network Analyzer ZNB40 (Rohde & Schwarz GmbH). The antenna realized gain was measured by the antenna measurement system with a full anechoic chamber in IoT center, Incheon, South Korea. The antenna measurement system includes PNA network analyzer E8362B (Agilent Technologies, Inc), microwave system amplifier 83017A (Keysight Technologies, Inc), and power supply 87421A (Keysight Technologies, Inc). Gain-transfer method was used.

## Electronic supplementary material

Supplementary Information
