# Supplementary material for: Reconfigurable Yagi-Uda antenna based on a silicon reflector with a solid-state plasma
Source: Sci Rep. 2017 Dec 8;7:17232. doi: 10.1038/s41598-017-17425-8 (PMC5722829; doi:10.1038/s41598-017-17425-8)
Supplement: Supplementary file 1 — Supplementary Information [file 41598_2017_17425_MOESM1_ESM.doc]

**Supplementary Information**

**Reconfigurable Yagi-Uda antenna based on a silicon reflector with a solid-state plasma**

*Da-Jin Kim,^1†^ Jang-Soon Park,^2†^ Cheol Ho Kim,^3†^ Jae Hur,^1^ Choong-Ki Kim,^1^* *Young-Kyun Cho,^3^ Jun-Bong Ko,^2^ Bonghyuk Park,^3^, Dongho Kim^2,a)^ and Yang-Kyu Choi,^1,a)^*

† These authors equally contributed to this work

^1^School of Electrical Engineering, Korea Advanced Institute of Science and Technology, (KAIST) 291 Daehak-ro, Yuseong-gu, Daejeon 34141, Republic of Korea

^2^Deparment of Electrical Engineering, Sejong University, 209 Neungdong-ro, Seoul 05006, Republic of Korea

^3^Wireless Home Network Research Team, Electronics and Telecommunications Research Institute, 218 Gajeong-ro, Yuseong-gu, Daejeon 34129, Republic of Korea

1. Authors to whom correspondence should be addressed.

Email addresses: dongkim@sejong.ac.kr and [ykchoi@ee.kaist.ac.kr](mailto:ykchoi@ee.kaist.ac.kr)

**Radiation patterns of the proposed reconfigurable Yagi-Uda antenna**

Figure S1 shows the radiation patterns of the proposed reconfigurable Yagi-Uda antenna obtained through the antenna simulator. Here, the conductivity of the top silicon (*σ_si_*) is assumed to be 10^5^ S/m and 0.01 S/m in the on-state and off-state. The model we used in the antenna simulator is described in Figure S1(a). The 2D radiation pattern on an E-plane at 5 GHz is shown in Figure S1(b). As the plasma silicon reflector is turned on, the front-realized gain (at 0°) increases and the rear-realized gain (at 180°) decreases, which proves the proposed Yagi-Uda antenna well control the directivity of the antenna. The 3D radiation patterns of the antenna when the plasma silicon reflector is in the off-state or on-state are shown in Figure S1(c) and (d).


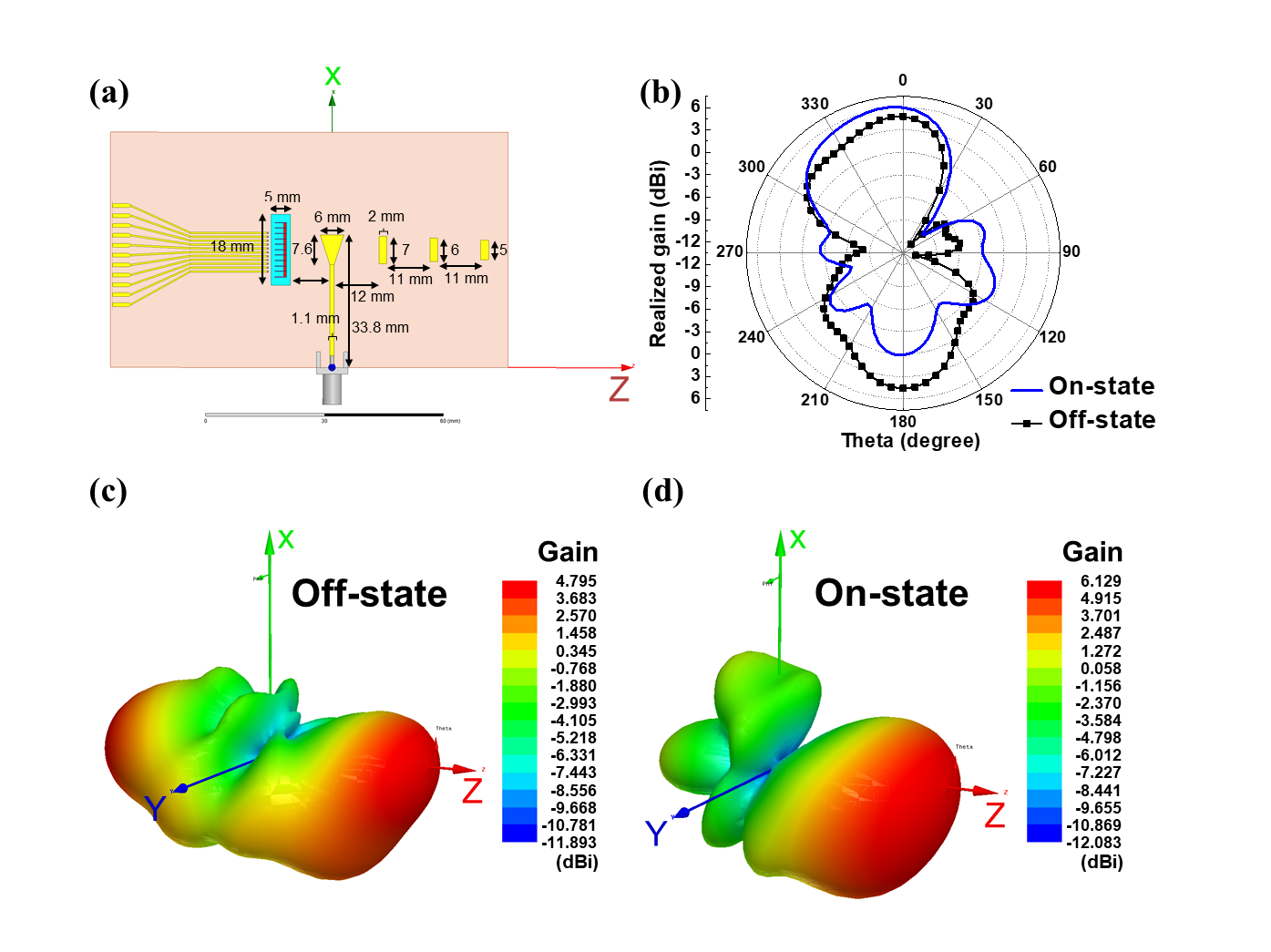


**Figure S1.** (a) The antenna model used in the simulator. (b) The 2D radiation pattern of the proposed Yagi-Uda antenna. (c) The 3D radiation patterns of the antenna in the off-state and (d) on-state.

**Antenna characteristics according to the thickness of the top silicon**

Figure S2 shows the influence of the thickness of the top silicon (*T_si_*) on antenna characteristics. Here, the conductivity of the top silicon (*σ_si_*) is assumed to be 50000 S/m in the on-state with an intrinsic channel length (*L_i_*) of 110 μm and a channel width (*W)* of 800 μm, and *T_si_* varies from 5 μm to 9 μm. Due to the low *σ_si_*, the antenna input reflection coefficient (S_11_) and the gain in the positive z-direction are insensitive to *T_si_*. Meanwhile, the power consumption decreases with a thinner *T_si_* because the current is rduced as *T_si_* decreases at the given applied voltage. Therefore, a thinner *T_si_* is preferred for the plasma silicon reflector.

**Figure S2.** Change of S_11_ and the gain in the positive z-direction according to *T_si_*.

**Antenna characteristics** **according to the conductivity of the bottom silicon**

Figure S3 shows the influence of the conductivity of the bottom silicon on antenna characteristics. Here, *σ_si_* is assumed to be 10^6^ S/m and 0.01 S/m in the on-state and off-state while *L_i_* is 110 μm, *W* is 800 μm, and *T_si_* is 5 μm. Simulation is conducted when the channel is in the on-state or off-state while the conductivity of the bottom silicon (*σ_bot_*) changes from 0.01 S/m to 100 S/m. As *σ_bot_* increased, resonance frequencies in the on-state and off-state become closer to each other, which means that it is harder to distinguish the on-state from the off-state with high *σ_bot_* (or vice versa). Similarly, as *σ_bot_* increases, the difference in the gain in the positive z-direction between the on-state and off-state also decreases. These results confirm that *σ_bot_* should be kept as low as possible to discriminate each state distinctively, so that the highest gain difference from each state can be obtained.

**Figure S3.** Change of S_11_ and the gain in the positive z-direction according to *σ_bot_*.

**Antenna characteristics according to the channel width of the p-i-n diode**

Figure S4 shows the influence of the channel width (*W*) of the p-i-n diode on antenna characteristics. Here, *σ_si_* is assumed to be 10^6^ S/m and 0.01 S/m in the on-state and off-state while *L_i_* is 110 μm, *T_si_* is 5 μm, and *σ_bot_* is 0.01 S/m. Simulation is conducted with *W* varying from 100 μm to 800 μm. As *W* decreases, the resonant frequency shifts and the gain in the positive z-direction decreases. Therefore, a larger *W* is preferable in a plasma silicon reflector.

**Figure S4.** Change of S_11_ and the gain in the positive z-direction according to *W*.

**Distribution of carrier concentration** **in the intrinsic channel region**

Figure S5 shows the distribution of carrier concentration in the intrinsic channel region according to *L_i_*. Here, the simulation is conducted for the following conditions: the applied voltage is 1.5 V, *T_si_* is 5 μm, *T_box_* is 1 μm, *W* is 1 μm, *L_c_* is 10 μm, and both *L_p_* and *L_n_* are 20 μm. The simulation is carried out with *L_i_* varying from 50 μm to 140 μm. The carrier concentration is the sum of the electron concentration and the hole concentration. The carrier concentration is uniformly distributed along the vertical direction regardless of *L_i_*. The carrier concentration is also almost uniformly distributed along the lateral direction.


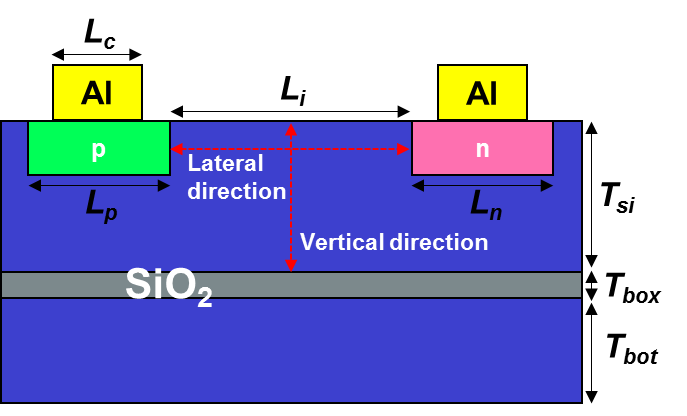


**Figure S5.** Distribution of the carrier concentration in the intrinsic channel region according to *L_i_*.

**Influence of gold wires on antenna characteristics**

Figure S6 shows the influence of gold wires connecting the plasma silicon reflector to the DC-bias lines on antenna characteristics. Here, *σ_si_* is assumed to be 0.01 S/m in the off-state while *L_i_* is 110 μm, *T_si_* is 5 μm, *W* is 800 μm, and *σ_bot_* is 0.01 S/m. The simulation is conducted in both cases whether the gold wires exist or not when the channel is off-state. As mentioned above, in Figures. S2 and S3, many gold wires also contribute to moving S_11_ toward lower frequencies. Similarly, the gain in the positive z-direction with gold wires is higher than without gold wires. The peak of the gain at 5 GHz can also be explained by the existence of gold wires.

**Figure S6.** Change of S_11_ and the gain in the positive z-direction according to the existence of gold wires
